# Influence of alcohol treatments on properties of silk-fibroin-based films for highly optically transparent coating applications

**DOI:** 10.1039/d0ra02634d

**Published:** 2020-04-21

**Authors:** Supranee Kaewpirom, Siridech Boonsang

**Affiliations:** Department of Chemistry, Faculty of Science, Burapha University Chonburi 20131 Thailand; Department of Electrical Engineering, Faculty of Engineering, King Mongkut's Institute of Technology Ladkrabang Bangkok 10520 Thailand siridech.bo@kmitl.ac.th +66 38 393 494 +66 38 103 066

## Abstract

Thin films of silk fibroin were prepared by solvent evaporation from calcium chloride/ethanol aqueous solution. The influence of alcohol treatments on thermal, mechanical and optical properties of silk-fibroin-based film is presented. To understand the conformal structure of the alcohol-treated silk fibroin film, the IR spectral decomposition method is employed. The optical properties especially the optical transparency, haze and fluorescence emission of alcohol-treated silk fibroin film is systematically investigated together with the conformal structure to understand the effect of the fibril such as the beta-sheet influencing the optical properties. Monohydric alcohol treatment increased beta-turn content in the regenerated silk fibroin structure. These affected the amount of light diffusion and scattering within silk-fibroin films. With alcohol-treatment, all the silk-fibroin films exhibit exceptional optical transparency (>90%) with different levels of optical haze (2.56–14.17%). In particular, ethanol-treated silk-fibroin films contain the highest content of beta-turns (22.8%). The ethanol-treated silk-fibroin films displayed a distinct interference of oscillating crests and troughs in the UV-Vis transmittance spectra, thereby showing the lowest optical haze of 2.56%. In contrast, the silk-fibroin films treated with methanol and propanol exhibit the highest (14.17%) and second-highest (10.29%) optical transmittance haze, respectively. The beta-turn content of the silk-fibroin films treated with methanol is the lowest (20.5%). These results show the relationship between the beta-turn content and optical haze properties. The results manifestly provide a method to manufacture exceptional optically transparent silk-fibroin films with adjustable light diffusion and scattering which can be designed to meet specific applications with the potential to provide UV-shielding protection *via* monohydric alcohol treatment.

## Abstract 2

This research presents a method to manufacture optically transparent silk-fibroin films with adjustable light diffusion and scattering *via* alcohol treatment.

## Graphical abstract

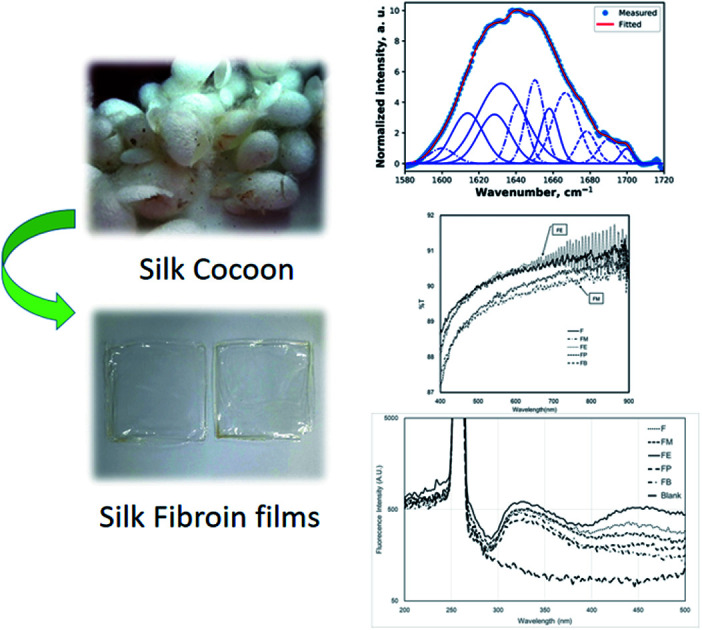

## Introduction

Silk fibroin is a biopolymer extracted from Thai silkworm cocoons by subtraction of sericin using a process called degumming. Silk fibroin has been extensively used, owing to its biocompatibility, optimal strength, bio-sustainability, biodegradability, and good processability in many forms such as films,^1^ gels,^2^ and 3D microperiodic scaffolds.^3^ Silk fibroin has become of interest for many applications including organic light-emitting diodes, organic field-effect transistors and organic photovoltaic cells.^4^

In the optoelectronics industry, plastic or glass substrates are mainly used for flexible electronic devices owing to their optical characteristics especially the transparency, mechanical strength, and desired processing temperature. Nevertheless, with the mass production of commercial electronic devices and their wide range applications in our everyday routine, they create a large amount of toxic and non-degradable electronic waste. To succeed this constraint, recently, products derived from silk fibroin have been developed as a promising substitute for existing plastic and glass substrates due to their environmental friendliness. Most possible applications of silk fibroin for flexible optical and electronic devices are focused on bio-integrated electronics, decomposable electronics, bio-sensing, and so on.^5,6^

In terms of optical properties, most of the aforementioned applications employ the relatively high transparency of silk-fibroin films. Though, for photovoltaic applications, in addition to the high optical transparency, the high optical haze is equally important. Optical haze is the proportion of the forwarding transmitted light, diffusely scattering. The increased scattering light within films can theoretically increase solar cell efficiency, and it is required for photovoltaic applications.^7^ Consequently, optical films preserving both high transparency and high optical haze concurrently are beneficial for solar cell applications. On the contrary, although optical haze is the preferred characteristic to be maximized in transparent substrates integrated into solar devices, other optoelectronic devices require distinct levels of light scattering. For example, display and touch screen devices demand films which can maintain great transparency with low optical haze. Consequently, to design the silk-fibroin film to meet the particular applications, the knowledge of the factor influencing the optical properties is necessarily required.

In this paper, the study of the influence of alcohol treatments on properties of silk-fibroin films, mainly for the high optical transparent coating applications, is presented. Silk fibroin is isolated from *Bombyx mori* cocoons. The thermal, mechanical and chemical characterization of silk fibroin is also revealed. To understand the conformal structure of the alcohol-treated silk-fibroin film, the IR spectra decomposing method is employed. The optical properties especially the optical transparency, haze and fluorescence emission of alcohol-treated silk-fibroin films is systematically investigated together with the conformal structure to understand the effect of the fibril compositions such as beta-sheet, beta-turn, alpha-helix and random coil, influencing on the optical properties.

## Experimental

### Materials

White *Bombyx mori* cocoons were from Udon Thani province, Thailand. Na_2_CO_3_ and CaCl_2_ were purchased from Ajax Finechem, and methanol, ethanol, *n*-propanol and *n*-butanol were purchased from QREC. All chemicals are analytical grade and were used as received without any further purification.

### Preparation of silk fibroin

The distilled water of 1.8 L was brought to the boil. Then 3.6037 g of Na_2_CO_3_ was put into the boiling water. After the solution was re-boiled, small pieces of white *Bombyx mori* cocoons (5 g) were added into the boiling Na_2_CO_3_ aqueous solution. The mixture was stirred occasionally for 30 min to ensure completed degumming process. The degummed silk was washed with distilled water until neutral and dried in a hot air oven at 60 °C for 24 h. Further drying was performed in a vacuum oven at 40 °C overnight. Dissolution of silk fibroin was carried out by the modification of Ajisawa's method^8^ as following. The degummed silk fibroin (1.00 g) was dissolved in 5.00 mL of CaCl_2_/EtOH/H_2_O solution (mole ratio of 1 : 2 : 8) at 110 °C for 2 h and then dialyzed against deionized water at room temperature for 48 h, using cellulose dialysis bag with molecular cut-off (MWCO) of 12 000–14 000 Da (Cellu Sep®). The deionized water was replaced every 4–6 h. The resulting mixture was centrifuged at 2200 rpm at room temperature (30–32 °C) for 20 min to remove impurities. A homogeneous silk fibroin solution was then gained. The final concentration of such silk fibroin solution, determined by the gravimetric method, was approximately 5.0% w/v.

### Preparation of silk fibroin film

Silk fibroin solution was mixed with various monohydric alcohols, as shown in Table 1. The polarity values are listed according to the literature.^9^ The volume ratio of silk fibroin solution and aqueous alcohol solution (10% v/v) was fixed at 3 : 1. After well mixing, 7.50 mL of the mixture was poured into a polyethylene terephthalate mold (8.7 × 8.7 cm^2^) and left to dry at room temperature. After drying at room temperature for 48 h, the film was further dried in a vacuum oven at room temperature for another 4 h to remove the residue water molecules.

**Table 1:** The detail of various monohydric alcohols used to induce the physical crosslinking in silk fibroin solution

Sample name	Alcohol solution	Number of carbon chain	Ratio of alcohol polarity	Polarity (*E*^N^_T_)
F	—	—	—	—
FM	Methanol	1	1.00 (1 : 1)	0.762 (ref. 9)
FE	Ethanol	2	0.50 (1 : 2)	0.654 (ref. 9)
FP	*n*-Propanol	3	0.33 (1 : 3)	0.617 (ref. 9)
FB	*n*-Butanol	4	0.25 (1 : 4)	0.586 (ref. 9)

### Conformational structure analysis by FTIR

The functional groups presented in the silk-fibroin film were analyzed by FT-IR spectrophotometer (PerkinElmer Frontier™ FT-IR/NIR system), equipped with two temperature-stabilized DTGS (deuterated triglycine sulfate) detectors, and a multiple-reflection ATR attachment. For each measurement, 4 scans were co-added with a resolution of 4 cm^−1^, and wavenumber ranged from 400 to 4000 cm^−1^. Curve fitting of the resulting FTIR spectra was performed covering the Amide I region (1580–1720 cm^−1^). The beta-sheet conformations were quantified from the area under the assigned peaks of 1616–1637 cm^−1^ regions and 1697–1703 cm^−1^ regions. Tyrosine side chains/aggregated strands (1616–1621 cm^−1^), random coils (1638–1655 cm^−1^), alpha helices (1656–1662 cm^−1^) and beta turns (1663–1696 cm^−1^) were also computed from the area under the assigned peaks.

### Insoluble gel fraction

Gel fraction of a silk-fibroin film was analyzed by the gravimetric method. Before the investigation, the silk-fibroin film with the area of 2.54 × 2.54 cm^2^ was dried at 60 °C for 48 h and weighed (*W*_1_). Then the dried film was immersed in deionized water at room temperature (30–32 °C) for 48 h to dispose of any soluble part. The lasting solid film was dried at 60 °C for 48 h and reweighed (*W*_2_). The gel fraction was calculated from the percentage ratio of the remaining solid weight and the initial weight of the dried film using eqn (1):1
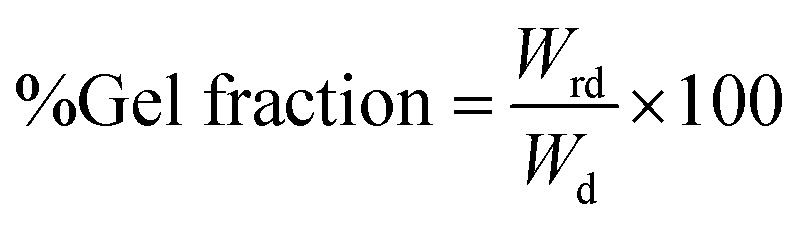


### Mechanical properties

Mechanical properties of the silk-fibroin films were evaluated on a universal tensile tester (Testometric, Micro 350), with a 500 N load cell. A silk-fibroin film was cut into 0.5 mm-wide and 5.0 mm-long strips and their thickness was measured using a digital micrometer. Grip separation and a cross-head speed were set at 30 mm and 10 mm min^−1^, respectively. The tests were carried out at 23 °C and 55% RH. At least 10 specimens were tested and the averaged values of Young's modulus, tensile strength and elongation at break (% *E*) were reported.

### UV/Vis analysis

A silk-fibroin film was measured for its transmittance over the visible-to-near-infrared region, from 380 nm to 780 nm, using UV/Vis/NIR spectrophotometer (PerkinElmer, LAMBDA 950). The spectrophotometer was fitted with a single monochromator, supplying low noise performance across a wide wavelength range. Data were collected by integrating sphere detector and plotted using the program and computer data station provided by the manufacturer. UV/Vis spectra were recorded using a 2.54 × 2.54 cm^2^ silk-fibroin film sample. The baseline measurement was accomplished automatically by the spectrophotometer with air as a reference.

### Optical haze measurement

The optical transmittance and the optical haze were measured by UV-Vis-NIR spectrophotometer (PerkinElmer LAMDA 950 with Integrating sphere detector). The reference standard is ASTM D 1003-00 Standard test method for haze and transmittance of transparent plastics.

### Fluorescence spectroscopy

A Shimadzu spectrofluorophotometer RF-6000 was used to record the fluorescence spectra of a silk-fibroin film. Fluorescence excitation-emission matrices were measured using an excitation wavelength of 256 nm. The corresponding emission in the 250–600 nm range with 5 nm increment was recorded. These wavelength ranges supported for intrinsic fluorescence measurements of tyrosine, tryptophan and other endogenous fluorophores in the UV-Vis range. The photomultiplier tube detector gain was fixed at 700 V.

## Results and discussion

### Mechanical characterization

**Fig. 1:**
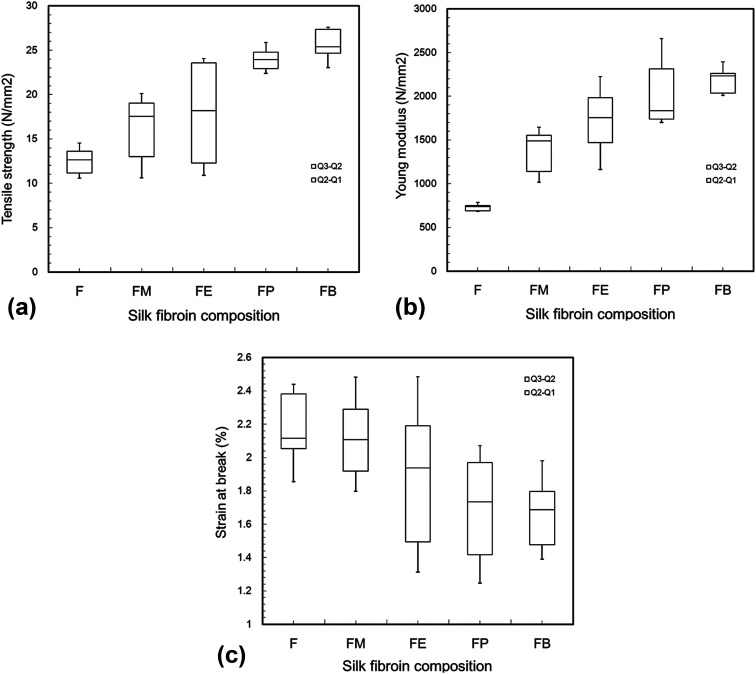
Mechanical properties (tensile strength (a), Young modulus (b), strain at break (c)) of silk fibroin films cross-linked by various monohydric alcohols.

Mechanical properties *e.g.* tensile modulus, tensile strength, and elongation at break of silk-fibroin films were analyzed through tensile testing and the results are shown in Fig. 1. As mentioned earlier, mechanical properties of silk fibroin depend significantly on its molecular structure components *e.g.* β-sheet crystallites and amorphous domain. The results from FTIR confirmed that the peaks at 1618 and 1512 cm^−1^, representing β-sheet conformation of Amide I and II, show relatively higher intensities than that of the peak at 1229 cm^−1^, accredited to random coil deformation of Amide III. These results suggest that the silk-fibroin film is predicted to show strong inter-chain interactions, and possesses superior strength. As expected, the silk-fibroin film shows low elongation at break of 2.26 ± 0.19% with high Young's modulus of 0.75 ± 0.09 GPa and moderate tensile strength of 12.6 ± 1.09 MPa. This was because the β-sheet crystallites blocked the molecular chains from sliding pass each other. Consequently, the breaking stress and Young's modulus were high and those values are following the values reported in literatures.^10–12^ Moreover, the proposed silk-fibroin film exhibits superior mechanical properties compared with a typical membrane from pure silk fibroin nano-fibrils that have ultimate stress at ∼13 MPa and strain at break of ∼1.35%.^13^

When monohydric alcohol was added, it is well known that the polarity of the alcohol caused some degree of conformational transition of silk fibroin solution from a random coil to β-sheet crystallization. The resulting crystallization produced physical crosslinks in the silk structure and the silk fibroin gel formed. According to Um *et al.*,^14^ the crystallization mechanism has been proposed that polar groups of alcohols attracted the water from silk fibroin molecules, resulting in the increased aggregation of hydrophobic amino acids, especially Gly and Ala, in the interior structure of silk fibroin molecules. In other words, the polarity of alcohol was an important factor that regulated the β-sheet crystallization as well as the crosslink density of the silk fibroin hydrogel. It was also reported by Kaewprasit *et al.*^15^ that as the length of carbon-chain of monohydric alcohol increased from 1 to 4, the ratio of polarity reduced from 1.00 to 0.25 (Table 1), resulting in elevated hydrophobic interaction between alcohol and silk fibroin molecules. Hence, *n*-butanol promoted the most rapid gelation of silk fibroin *via* β-sheet crystallization. In this present study, it was evidenced by the increase in tensile strength (from 18.4 ± 1.2 to 26.0 ± 1.6 MPa) and Young's modulus (from 1.5 ± 0.1 to 2.2 ± 0.1 GPa) of the silk hydrogel films as the length of the carbon-chain of alcohol increased from C1 to C4. Contrariwise, the elongation at break decreased from 2.13 ± 0.15 to 1.67 ± 0.10%. Convincingly, the addition of monohydric alcohol into a silk fibroin solution gives rise to an increase in mechanical strength of the silk-fibroin film.

### Gel fraction

**Fig. 2:**
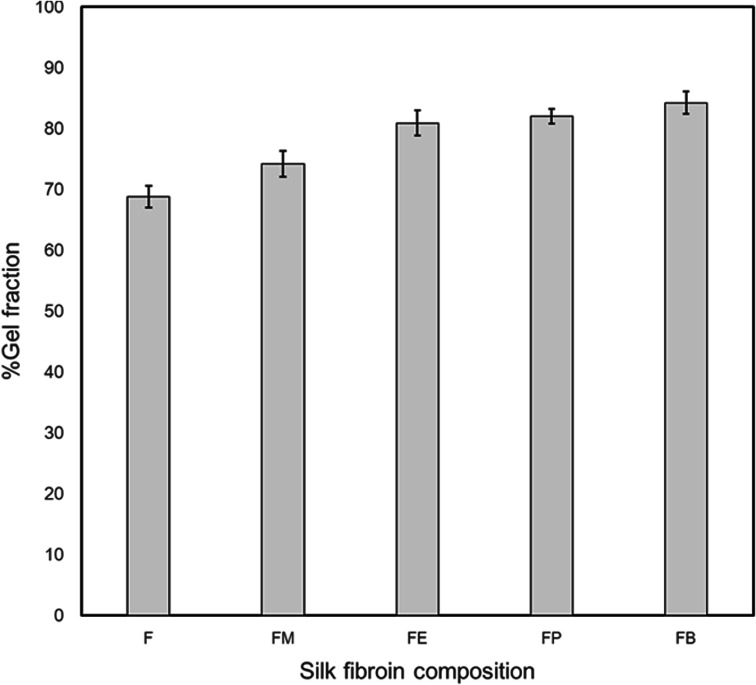
The percentage of gel fraction for dried silk fibroin films with 10 wt% various monohydric alcohol addition after immersion in deionized water at room temperature (30–32 °C) for 2 days.

Gel fraction was used to measure the insoluble part of silk-fibroin films after immersion in deionized water at room temperature (30–32 °C) for 48 h and the percentage of gel fraction is shown in Fig. 2. Without the addition of alcohol, silk-fibroin film (F) showed the gel fraction of 68.8 ± 1.7%. This value was significantly lower than those of the silk-fibroin films with the addition of monohydric alcohols (74.2 ± 2.1% for FM, 80.9 ± 2.0% for FE, 82.0 ± 1.2% for FP, and 84.3 ± 1.9% for FB). Moreover, it was also found that the gel fraction increased with an increasing amount of carbon atom in monohydric alcohol. This finding was in good accordance with the mechanical strength of the silk-fibroin films that also increased with an increasing amount of carbon atom in monohydric alcohol. This could be related to the conformational structure of the silk fibroin itself after monohydric alcohol was evaporated. As we mentioned earlier, the polarity of the alcohol caused some degree of β-sheet crystallization that promoted physical crosslinks in the silk structure, resulting in gel formation. Therefore, the longer the carbon-chain of a monohydric alcohol, the lower the polarity of the alcohol is, resulting in the higher degree of physical crosslink in the silk structure.

### Conformational structure characterization

Silk consists of two main proteins, sericin and fibroin. According to Fraser and MacRae,^16^*Bombyx mori* silk fibroin comprises of a high proportion of three α-amino acids, glycine (Gly, 45%), alanine (Ala, 29%), and serine (Ser, 12%), with the molar ratio of 3 : 2 : 1. Fibroin is the structural core of silk, while sericin is the viscous material enclosing it. Fibroin principally composes of the amino acids in the sequence Gly–Ser–Gly–Ala–Gly–Ala and develops beta-pleated sheets, β-keratin, *via* hydrogen bonds. Consequently, β-sheets are commonly denoted as crystallites that play an important role in silk's mechanical properties.^4^

**Fig. 3:**
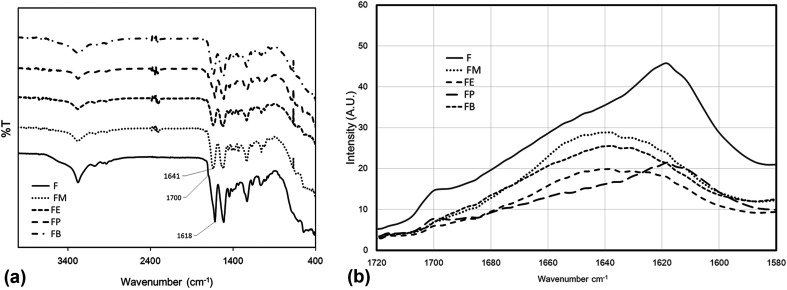
(a) ATR-FTIR spectra of silk fibroins treated with various monohydric alcohols. (b) ATR-FTIR spectra of the Amide I absorption band.

In this paper, silk fibroin was separated from silk cocoons by washing out sericin, the gum that coats the fibroins and allowing them to stick to each other, using Na_2_CO_3_ aqueous solution. FTIR spectra of silk fibroin were shown in Fig. 3(a). A broad absorption band between 3100 and 3700 cm^−1^ combined with a distinctive peak at 3278 cm^−1^ belongs to the stack stretching vibration of N–H and O–H of peptide groups. The C–H stretching peaks found at 3073, 2937, and 2926 cm^−1^ were due to C–H aromatic, C–H asymmetrical, and C–H symmetrical, respectively. The C–H_2_ scissoring was also found at 1444 cm^−1^. Sharp peaks at 1618, 1512, and 1229 cm^−1^ were assigned to Amide I, II (β-sheet conformation) and Amide III (random coil conformation), respectively. These are typical forms of peptides, as reported in literature.^15,17^

Among the amide modes of the peptide groups, the Amide I absorption band is commonly used as the key description of the secondary structure.^18^ The Amide I molecular vibration composes of peptide carbonyl stretch and displays the secondary structure sensitivity. Experimental frequency–structure relationships suggest that β-sheets have a strong absorption band at 1610–1680 cm^−1^ and a weaker band at 1640–1690 cm^−1^. The random coil and alpha-helix structure are normally found at 1640–1650 and 1650–1660 cm^−1^, respectively. Fig. 3(b) displays the expanded view (1580–1720 cm^−1^) of ATR-FTIR absorbance spectra of the silk-fibroin films with/without the monohydric alcohol treatment. The non-treated SF film comprised of beta-sheet and random coil structures are shown by a peak and a shoulder at 1620 cm^−1^ and 1645 cm^−1^, respectively. Only the spectra of the silk-fibroin film with *n*-propanol treatment exhibit similar absorption spectra to the non-treated SF film. The rest of the alcohol-treated silk fibroin spectra demonstrates the characteristic of the random coil and beta-turn dominated spectra.

For more detail analysis, Fourier-transform self-deconvolution (FSD) investigation of Amide I is performed to analyze the conformational structural content of silk fibroin films.^19,20^ The application of both Fourier-transform self-deconvolution and peak fitting is an established technique for interpreting spectra with overlapping bands, facilitating the semi-quantitative evaluation of the underlying components. Nevertheless, there is no fixed methodology for either process and they are subjected to noise in their separation of peak maxima and number. An approach to FTIR peak fitting was introduced and proposed to facilitate the fitting process without deconvolution and to decrease errors contributed by user manipulation.^21^ Curve-fitting by such a method was utilized in this work using Python library.^22^ Peak positions of the curve-fitted peaks were allocated to their potential conformations of secondary structure and side chains as described in Table 2.^20^

**Table 2:** Vibrational band assignments in the Amide I region for *Bombyx mori* silk fibroin^20^

Wave number range (cm^−1^)	Assignment
1605–1615	(Tyr) side chains/aggregated strands
1616–1621	Aggregated beta-strands/beta-sheets (weak)^a^
1622–1627	Beta-sheets (strong)^a^
1628–1637	Beta-sheets (strong)^b^
1638–1646	Random coils/extended chains
1647–1655	Random coils
1656–1662	Alpha-helices
1663–1670	Turn
1671–1685	Turn
1686–1696	Turn
1697–1703	Beta-sheets (weak)^a^

a Intermolecular beta-sheets.

b Intramolecular beta-sheets.

**Fig. 4:**
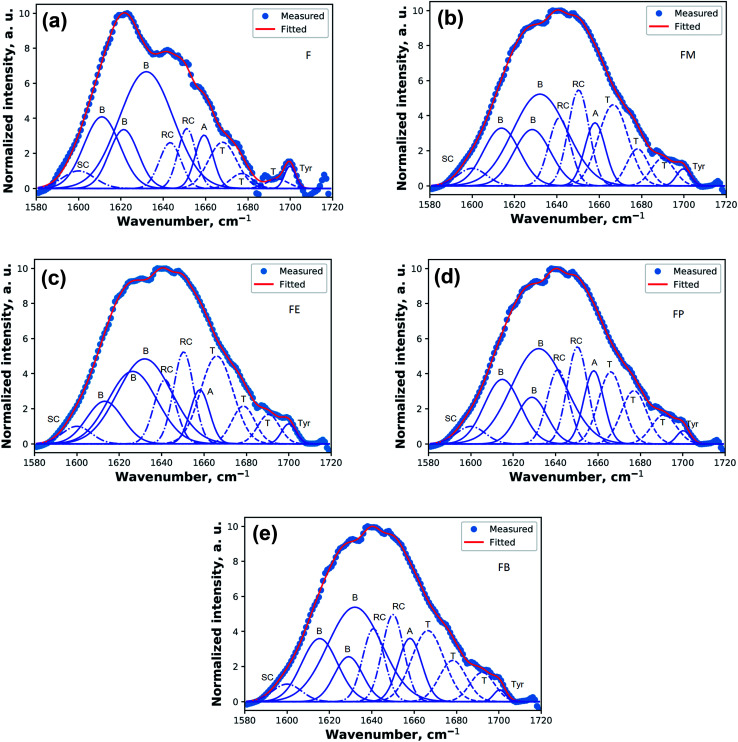
Absorbance Amide I spectra of silk fibroin films (a) with no alcohol treatment and treatment with (b) methanol (c) ethanol (d) *n*-propanol (e) *n*-butanol. The spectra shows the results after fitted with Gaussian profile. The solid line represents the deduced absorbance band. The dotted lines represent the contributions to the Amide I band and are marked as random coil (R), beta-sheets (B), alpha-helices (A), beta-turns (T), and side chains (SC).

Fig. 4 shows the resulted curves of FTIR absorbance spectra for Amide I, after fitting with Gaussian profile. The peaks are identified with abbreviations that represent beta turns (T), alpha-helix (A), random coil (R), beta-sheets (B), and side chains (SC). The dotted line is the measured absorbance spectra. The solid line is the summation of the individual contributions or the summation of the deconvoluted Gaussian component curves. Fig. 4(a) illustrates the fitted results of a silk-fibroin film without additional alcohol treatment. It displays the distinctive beta-sheets characteristic at 1619 cm^−1^. Besides, the fibroin film secondary structures can also be identified as beta-sheet, alpha-helix, beta-turn, and random coil with the relative amount of 66.6 ± 0.5%, 5.9 ± 0.8%, 11.3 ± 0.5%, and 12.7 ± 1.0%, respectively.

**Fig. 5:**
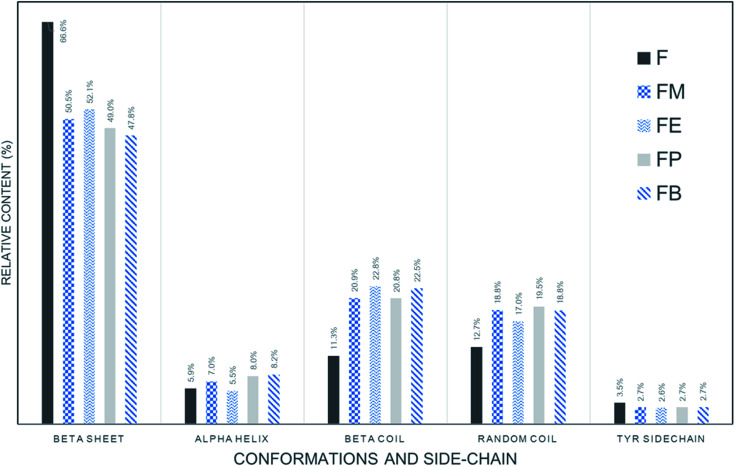
Relative contents of secondary structures in regenerated SF films prepared with determined by quantitative ATR-FTIR.

Fig. 4(b)–(e) show the fitted results of the silk-fibroin films with the addition of monohydric alcohols. Fig. 5 shows the summary of the secondary structure relative content of SF films with/without additional alcohol treatment. It is apparent when adding various monohydric alcohols, the beta-sheet content of each alcohol-treated silk-fibroin film was noticeably lower than that of the non-treated SF film. Also, the tyrosine side chain and alpha-helix contents of all the SF films were almost unaltered by additional alcohol treatment. Nevertheless, the beta-turn and random coil structures of the silk fibroin film was found to be noticeably higher while monohydric alcohols were added. The increase in beta turn content resulted from the hydrophobic interaction between silk fibroin protein chains and additional alcohols. The increment in the length of the carbon chain of alcohol solution induced the expansion of the beta-sheet content in the hydrogels.^15^ In agreement with Braun and Viney,^23^*Bombyx mori* silk fibroin monomer comprises the ratio of hydrophobic to polar residues (H : P) at 0.79 : 0.21. The hydrophobic residue is recognized as the basic element of beta-sheet structure and represents the crystalline component of silk fibroin.^24,25^ When various monohydric alcohols were combined into a silk fibroin solution, the number of both hydroxyl group and carbon-chain directly influenced the polarity of the alcohols (Table 1). The ratio of the polarity of monohydric alcohols was decreased from 1.00 (1 : 1) to 0.25 (1 : 4) when the length of carbon-chain of the alcohol increased from 1 to 4. With the reduction of its polarity, the water solubility of alcohol also diminished. Consequently, the low polar alcohol might associate with silk fibroin *via* hydrophobic interaction, which is the fundamental impetus for the beta-sheet creation of the silk fibroin segment through hydrogen bonds. Likewise described by Kasoju *et al.*,^26^ the addition of polar protic organic solvents such as methanol, ethanol, isopropanol and *n*-butanol to the fibroin–water mixture disrupted the hydrophobic hydration of regenerated silk fibroin, produced the conformational alterations in regenerated silk fibroin that eventually directed to its aggregation and consequent gelation. In other words, polar protic organic solvents can provoke the self-assembly of less-ordered randomly coiled fibroin molecules into ordered crystalline β-sheet aggregates.^27^

### Optical transmittance and haze

**Fig. 6:**
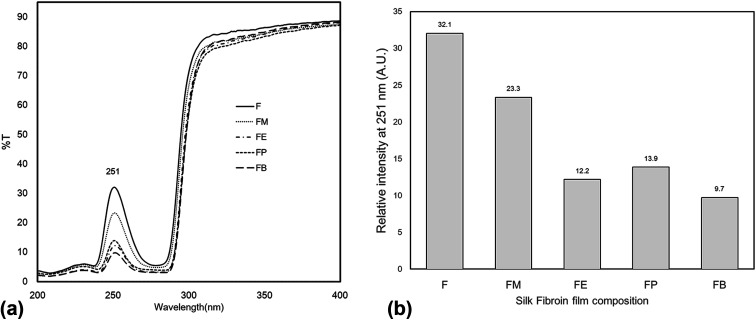
UV-Vis spectra of silk fibroin film in different composition (a) in the UV range of 200–400 nm (b) the relative intensity of the UV transmittance at the peak of 251 nm.

To our knowledge, the outstanding transparency characteristic of the silk-fibroin film makes this material applicable to various ultra-transparent applications. Fig. 6 shows transmittance spectra collected from the silk-fibroin film with ≈10 μm thickness to evaluate the transparency in the UV-visible spectral range (200–900 nm). The results reveal that the film exhibited excellent transmission from 350 nm to 900 nm (visible range), where the percentage transmittance of approximately 90% was observed. Interestingly, the film can absorb UV light in the spectral range of 200–300 nm. A point worth noting is that the silk fibroin fabricated by solvent casting displayed an excellent transmission property with the potential to UV-shield and highly preserve the intrinsic optical property of the substrate onto which it may be mounted. The principal chromophores absorbing in the UV region are considered to be the aromatic amino acids, tyrosine, phenylalanine and tryptophan, which are present in the silk structure.^17^ This corresponds to the tyrosine content found in dried silk-fibroin films (Fig. 5).

Fig. 6(a) shows the experimental results of UV-Vis spectra of the silk-fibroin films with/without the addition of monohydric alcohols. The transmission spectra of the regenerated silk fibroin in solution displayed a wide peak in the region 240–280 nm. The principal chromophores absorbing in the UV region are likely the aromatic amino acids, tyrosine, phenylalanine and tryptophan, which are present in the silk chain Fig. 6(b) summarizes the peak value of the transmission spectra in the UV region (200–280 nm) for each of the silk-fibroin film composition. These results are associated with the tyrosine side chain content in the SF films as shown in the previous section (Fig. 5).

**Fig. 7:**
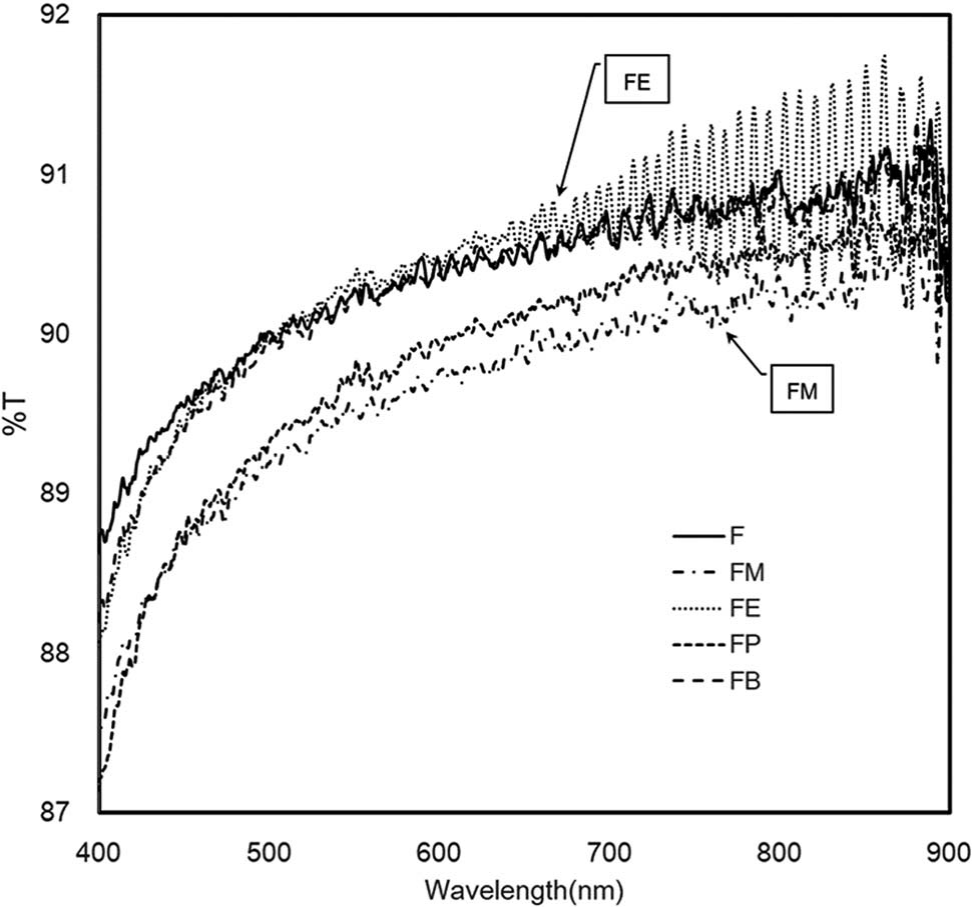
UV/Vis spectra of silk fibroin films showing outstanding UV shield and optical transparency.

Fig. 7 shows the UV-Vis transmission spectra (400–900 nm) of the silk-fibroin films with different contents of secondary structure. All of the alcohol-treated films exhibit exceptional optical transparency. In the overall range of spectra, the results from the bare silk-fibroin films (indicated as F) reveals the comparatively the same performance as the silk fibroin treated with ethanol, FE, and with n-butanol, FB. Particularly, in the case of FE, the spectra show a distinct interference of oscillating crests and troughs possibly coming from the multiple reflections of both sides of film surfaces. By analysis the interference pattern of the spectra, it allows us to determine the thickness of the layers by measuring the distance between the fringes. The interference pattern exhibits a good homogeneous film with a thickness of 7.5 ± 0.2 μm. Besides, it is well known that this interference pattern occurs only when the film has the characteristic of a highly smooth and flat surface with very low measured diffuse transmittance. The plausible factor that may contribute to such outstanding properties is the high beta-turn content in the silk-fibroin films (22.8% and 22.5% for FE and FB respectively). The beta-turn may benefit the alignment of the beta-sheet structure so that when the incident light directly passing through the beta-sheet crystalline structure, it continues to propagate through with the minimal scattering and diffusion.

To confirm the above conclusion, the optical haze measurement was performed. Table 3 shows the optical haze measurement results of the silk-fibroin films with/without the addition of monohydric alcohols. The silk-fibroin films treated with methanol and propanol exhibits the highest and second highest optical transmittance haze, respectively. The beta-sheet structures in silk fibroin can grow nano-fibres with a length about nanometers, which are expansively dispersed throughout the thin silk-fibroin film. These fibril structures can efficiently scatter light and manifestly intensification the optical haze. However, it was noticeably presented in Table 3 that % haze of silk-fibroin films treated with ethanol and *n*-butanol is significantly lower than that of FM and FP films. Therefore, the deduction from the optical haze measurement is that the light diffusion and scattering within the FE and FB film is substantially minor. These results confirm the effect of the beta-turn contents (as shown in Fig. 5) on the light diffusion and scattering within silk-fibroin films as discussed earlier in the optical transmittance results.

**Table 3:** The optical haze measurement results of the silk fibroin films with/without the addition of monohydric alcohols

Sample name	Haze (%)	Diffuse luminous transmittance (%)	Light transmittance (%)	Sample and instrument scatter	Instrument scatter	*K*
F	10.9	12.76	90.46	12.79	3.24	0.0222
FM	14.17	15.78	90.39	15.81	3.33	0.0222
FE	2.56	5.35	91.21	5.38	3.34	0.0222
FP	10.29	12.33	90.45	12.36	3.38	0.0222
FB	4.62	7.2	90.84	7.23	3.34	0.0222

### Fluorescence emission

It is generally known that fluorescence spectra are predominantly influenced by the structure of proteins.^17^ Silk fibroin composes of a mixture of random-coil, the silk I and α-helical structures, according to the non-crystalline region of the protein. Since not all beta-sheet structure dissolves in solution, only a relatively small amount of beta-sheet silk II secondary formation is additionally present in the crystalline section. In case of a long silk chain, the dominance of tyrosine throughout the sequence is obvious, whereas tryptophan residues are available in minority. Furthermore, di-tyrosine cross-links are additionally confirmed to emit fluorescence in the 400 nm region.

To examine the protein structure and dynamics in various essential biochemical and biophysical investigations, the intrinsic fluorescence of proteins has been commonly employed. Spectral identities are subjective to the position of the fluorophores in the protein macromolecule and also the properties of the microenvironment. Tryptophan, normally presented in minority, has the greatest extinction coefficient and quantum yield among the three aromatic amino acids. Consequently, it is frequently utilized to determine protein structure. Georgakoudi *et al.* described a quantitative examination and modelling of the measured spectra as a combination of fluorescing biochemical components that exhibit important evidence with respects to the structure of the protein.^28^ They observed that the characteristic of the structural conformation of the silk fibroin protein is profoundly related to the fluorescence and excitation–emission spectra associated with tryptophan.

**Fig. 8:**
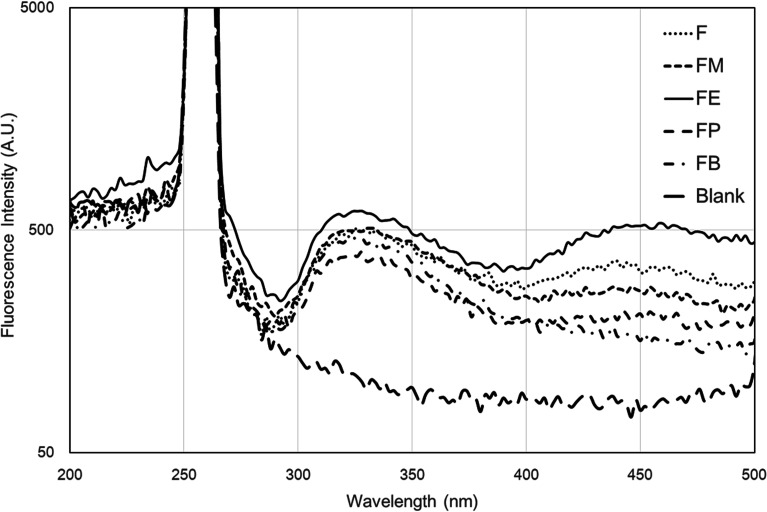
Fluorescence emission spectra of the differently alcohol-treated silk fibroin films.

Fig. 8 presents the fluorescence emission spectra acquired from the differently alcohol-treated silk-fibroin film samples. Excitation of a regenerated silk-fibroin film at 256 nm leads to fluorescence with emission in UV and visible region of the spectrum (Fig. 8). Two wavelength maxima of the fluorescence intensity for regenerated silk-fibroin films (after excitation at 256 nm) are recognized at 320 nm and 450 nm. The position of the emission maximum in the range of 310–345 nm is characteristic of tryptophan residues.^28^ It was also published by Vivian and Callis^29^ that the fluorescence intensity, wavelength maximum (*λ*_max_), of tryptophan is considerably sensitive to its local environment, ranging from ∼308 nm (azurin) to ∼355 nm (*e.g.*, glucagon) and approximately agrees with the degree of solvent exposure of the chromophore. In this present study, it was found that emission from the tryptophan component is also consistent with tryptophan presented in a highly hydrophilic environment that is comparable for all types of silk samples. Moreover, fluorescence emission recognised at the location of the emission maximum in the range 400–480 nm region is owing to di-tyrosine crosslinks.^28^ Tyrosine can be excited at wavelengths comparable to that of tryptophan but emits at a distinctly different wavelength. While tyrosine is less fluorescent than the tryptophan, it can produce a significant signal, as it is often present in large numbers in many proteins. In our study, only the silk-fibroin film (F) and ethanol-treated silk-fibroin film (FE) showed the emission peak at the wavelength maximum of ∼450 nm. To our knowledge, tryptophan is as an essential α-amino acid that contains an α-amino group, an α-carboxylic acid group, and a side chain indole, making it a non-polar aromatic amino acid. The indole group of tryptophan residues is the dominant source of absorbance and emission in protein. Beside, tyrosine is a non-essential amino acid with a polar aromatic side group. Hence, protein fluorescence can be used as a diagnostic of the conformational state of a protein.

## Conclusions

In summary, we present a detailed study of the influence of alcohol treatments on the properties of silk-fibroin films for highly optical transparent coating applications. The transparent silk fibroin solution was mixed with various monohydric alcohols namely, methanol (FM), ethanol (FE), *n*-propanol (FP) and *n*-butanol (FB). Mechanical properties *e.g.* tensile modulus, tensile strength, and elongation at break of silk-fibroin films were analyzed through tensile testing. The conformational structure characterization was also performed by analyzing the FTIR spectra with a deconvolution procedure. When adding various monohydric alcohols, the beta-sheet content of each alcohol-treated SF film was noticeably lower than that of the non-treated SF film. The beta-turn structure of the silk fibroin hydrogel was found to be noticeably higher when monohydric alcohols were added. The UV-Vis transmittance spectra were collected from the silk-fibroin film with 7.5 mm thickness to evaluate the transparency in the spectral range (200–900 nm). The results showed that the film exhibited excellent transmission from 350 nm to 900 nm (visible range) where the percentage transmittance of approximately 90% was observed. Particularly, in the case of FE silk-fibroin films, the spectra show a distinct interference of oscillating crests and troughs possibly coming from the multiple reflections of both sides of film surfaces. The plausible factor that may contribute to such outstanding properties is the high beta-turn content in the silk-fibroin films. These results were also confirmed by the optical haze measurement. Lastly, fluorescence emission spectra were acquired from the differently alcohol-treated silk-fibroin film samples. Excitation of regenerated silk fibroin at 256 nm leads to fluorescence with emission in UV and visible region of the spectrum. The maximum wavelengths of fluorescence intensity of regenerated silk fibroin samples (after excitation at 256 nm) were observed at 320 nm and 450 nm. Only the two silk-fibroin films, *e.g.* FE and F exhibited the emission wavelength ∼400 nm region.

## Conflicts of interest

There are no conflicts to declare.

## Supplementary Material
